# Mapping emotion regulation profiles in adolescence and examining their associations with mental health outcomes

**DOI:** 10.1186/s13034-026-01180-8

**Published:** 2026-09-25

**Authors:** Moa Nilsson, Elsa Segercrantz Walther, Linn Willkomm, Jonas Bjärehed, Daiva Daukantaitė

**Affiliations:** Department of Psychology, Lund University, Lund, 221 00 Sweden

**Keywords:** Adolescent’s Emotion Regulation Strategies Questionnaire – Extended (AERSQ-E), Emotion regulation, Emotion regulation strategies, Adolescents, Latent profile analysis, Emotion regulation profiles, Mental health, Person-centred approach

## Abstract

**Background:**

Emotion regulation (ER) plays a crucial role in adolescent mental health, yet its complexity is often overlooked. ER involves the use of multiple strategies, and individuals frequently combine several strategies to manage their emotions. To better capture this complexity, person-centred approaches have increasingly been used to identify ER strategy profiles. However, research on ER profiles among adolescents remains limited. The present study therefore adopted a person-centred approach to identify ER strategy profiles in adolescents and examine their associations with mental health outcomes.

**Methods:**

Survey data were collected from a community sample of Swedish adolescents (*n* = 759; *M*_age_ = 13.9 years; 48.6% girls). The survey included measures of six ER strategies, including rumination/negative thinking, positive reorientation, social support, aggressive outlet, creative expression, and distraction, as well as measures of life satisfaction, anxiety, depression, and non-suicidal self-injury (NSSI). Latent profile analysis was used to identify distinct ER strategy profiles.

**Results:**

Five ER profiles were identified: Positive Reorientation–Focused, Minimal Strategy, Multiple Strategy, Rumination–Focused, and Creative Rumination-Aggression. The profiles differed significantly across all mental health outcomes, with large effect sizes. The Positive Reorientation–Focused and Minimal Strategy profiles showed the most favourable mental health, whereas the Multiple Strategy profile showed intermediate outcomes. The Rumination–Focused and Creative Rumination-Aggression profiles showed the poorest mental health. Notably, the Minimal Strategy profile, comprising primarily boys, was associated with favourable outcomes despite limited strategy use. The Creative Rumination-Aggression profile, which consisted predominantly of girls, showed the most severe pattern, with nearly half of its members scoring within the clinical range for anxiety and 75.5% reporting repetitive NSSI. Creative expression occurred in profiles characterized by both favourable and poorer mental health, suggesting that its associations with mental health may vary depending on the broader ER configuration in which it occurrs.

**Conclusions:**

The findings suggest that favourable mental health outcomes are associated with multiple ER strategy configurations rather than a single optimal pattern, and that the associations between individual strategies and mental health may depend on the broader regulatory repertoire in which they are embedded. Longitudinal research is needed to clarify whether minimal strategy use reflects an adaptive pattern or a potential vulnerability over time, and to evaluate the relative stability and developmental relevance of the five- and six-profile solutions.

**Supplementary Information:**

The online version contains supplementary material available at https://doi.org/10.1186/s13034-026-01180-8.

Emotion regulation (ER) is a key psychological process for adolescents’ mental health, as individual differences in ER are systematically associated with a range of mental health outcomes. Effective ER is linked to social competence, resilience, and favourable mental health, whereas ER difficulties and maladaptive strategies such as rumination/negative thinking, defined as repetitive and self-focused thinking about distress [1], are associated with internalizing and externalizing problems, as well as non-suicidal self-injury (NSSI; e.g., [2–8]).

Much of the previous research on ER has used variable-oriented approaches that examine individual ER strategies (e.g., [9, 10]), or overall levels of regulatory difficulty (e.g., [1, 2, 6, 11]). While informative, such approaches may overlook the dynamic and context-dependent nature of ER. In everyday life, adolescents typically use multiple strategies that may interact with one another and vary according to situational demands and individual differences. To better capture the complexity of ER, a growing body of research has adopted person-centred approaches [12, 13] to identify distinct ER profiles based on the co-occurrence of multiple strategies or abilities within individuals (e.g., [14, 15]). However, research in this area remains limited, particularly in adolescence (for a review, see [16]), and is further constrained by several methodological limitations, including reliance on ER measures originally developed for adults (e.g., [17, 18]), modest sample sizes [13, 14], highly specific samples (e.g., adolescents engaging in unprotected sex, [19]) and a focus on specific emotions (e.g., anger, [10]), all of which limit generalizability. To address these limitations, the present study examines ER profiles using the Adolescents’ Emotion Regulation Strategies Questionnaire–Extended (AERSQ-E; [20]), a measure specifically developed for adolescents. Drawing on a large, community-based sample of Swedish adolescents, we aim to identify distinct ER strategy profiles and examine their associations with different mental health outcomes.

## Emotion regulation conceptualization

Emotion regulation (ER) refers to the processes involved in evaluating and modifying emotional responses in terms of their intensity, duration, and salience [21]. ER has been conceptualized from different theoretical perspectives, most commonly distinguished as ability-oriented and strategy-oriented approaches (e.g., [4]). Ability-oriented approaches define ER as the capacity to effectively manage and modulate emotional experiences and responses, viewing ER as a broader regulatory capacity involving skills such as emotional awareness, acceptance of emotions, and the ability to pursue goal-directed behaviour under emotional distress [22]. In contrast, strategy-oriented approaches emphasize the specific regulatory strategies (e.g., cognitive reappraisal, suppression, or rumination/negative thinking) individuals use and the habitual patterns with which these strategies are deployed across contexts [23, 24]. In the present study, we adopt a strategy-oriented approach, as it is particularly well suited to research with adolescents (e.g., [4]). During adolescence, regulatory capacities are still developing, and emotional experiences are often heightened and contextually sensitive [25, 26]. Focusing on the specific strategies adolescents use, rather than their overall regulatory ability, allows for a more precise examination of naturally occurring patterns of emotion management in everyday life [23, 24].

One of the most influential strategy-oriented frameworks is Gross’ [24] process model of ER, which describes how emotions can be regulated at different stages of the emotion generation process. In this model, ER involves a sequence of processes, including identifying the emotional state, selecting where to intervene, implementing regulatory strategies, and monitoring the outcomes of those strategies. According to this model, individuals may engage in antecedent-focused strategies (e.g., cognitive reappraisal) that modify emotional responses before they are fully activated, or response-focused strategies (e.g., suppression) that act after an emotion has been generated. By distinguishing strategies based on their timing and functional properties, the model highlights that regulatory strategies differ in their potential consequences. Importantly, the process model also implies that individuals may employ multiple regulatory strategies across different stages of the emotion generation process [24, 27]. Consequently, ER in everyday life is likely to involve combinations of strategies rather than reliance on a single strategy in isolation [23].

### Emotion regulation profiles in adolescence

To better capture the complexity of ER, researchers have increasingly adopted person-centred approaches [12, 13] that identify patterns of co-occurring ER strategies or abilities within individuals, and examine how these combine to form distinct ER profiles. However, relatively few studies have examined such profiles in adolescence. A recent review by Brandão [16] identified 38 ER profile studies, of which only four included adolescent samples, highlighting a substantial gap in the literature. The available evidence suggests considerable heterogeneity in adolescents’ ER patterns. Across studies, three to four ER profiles are generally identified, differing in overall regulatory engagement, the balance between adaptive and maladaptive strategies, and the breadth of adolescents’ regulatory repertoires. Previous studies have identified strategy-based profiles characterized by low engagement in regulatory strategies, mixed or moderate strategy use, or broader use of multiple strategies (e.g., [14, 28]). Otterpohl et al. [10] examined ER profiles within the specific domain of anger regulation and identified four profiles that differed in the balance between a priori-defined adaptive strategies (e.g., reappraisal and problem-solving) and maladaptive strategies (e.g., rumination/negative thinking and suppression), including profiles characterized by predominantly adaptive regulation, predominantly maladaptive regulation, and mixed patterns of strategy use.

Although these studies consistently point to heterogeneity in adolescents’ ER patterns, important methodological differences remain. Adolescents differ not only in how frequently they use ER strategies, but also in how these strategies cluster and co-occur within individuals. Studies vary in sample size, the ER instruments used, the regulatory domains examined, and the cultural contexts in which they are conducted. As a result, findings are not always directly comparable, and it remains unclear whether similar ER profiles can be reliably identified across different measures and populations, highlighting the need for further research.

### Emotion regulation and mental health

Building on the identification of distinct ER profiles, an important consideration is how these regulatory patterns relate to adolescent mental health. A substantial body of research demonstrates that ER difficulties are associated with the emergence and persistence of mental health problems [6, 18, 29–31]. This relationship is especially prominent during adolescence, a period characterized by increased emotional reactivity and a well-documented rise in symptoms of anxiety, depression, and NSSI [32–35].

When ER is examined in terms of individual strategies, consistent patterns emerge. Frequent engagement in rumination/negative thinking, suppression, avoidance, and aggressive outlet has been linked to higher levels of internalizing symptoms and increased risk of NSSI [1, 2, 6, 8, 11, 20, 36]. These associations are especially noteworthy in light of the documented increase in NSSI alongside rising mental health problems in recent decades [32]. Additionally, as proposed by the functional emotion regulation model of NSSI [37–40], NSSI can serve as a maladaptive means of regulating overwhelming negative affect when more adaptive strategies are ineffective or unavailable, for example, by functioning as a distraction from negative emotions such as sadness. In contrast, strategies such as cognitive reappraisal, problem-solving, acceptance, and positive reorientation are generally associated with more favourable emotional outcomes (e.g., [2–4, 8, 20, 41]). Building on these findings, profile-based studies have linked ER profiles to mental health outcomes. Lougheed and Hollenstein [14], for instance, found that adolescents with limited strategy repertoires reported higher levels of internalizing symptoms, whereas adolescents with broader use of strategies reported lower distress. Otterpohl et al. [10] showed that anger regulation profiles marked by greater reliance on rumination/negative thinking and suppression were associated with poorer psychosocial adjustment, while profiles including higher levels of reappraisal and problem-solving were linked to more favourable mental health outcomes. Similarly, Zhang et al. [28] demonstrated that a profile characterized by high levels of rumination/negative thinking and catastrophizing was associated with elevated negative affect and increased risk of NSSI, whereas a profile marked by greater positive reappraisal was associated with lower levels of distress. Together, these studies suggest that mental health outcomes are associated not only with the presence of specific ER strategies, but also with how strategies co-occur within individuals. Importantly, ER patterns and their associations with mental health do not develop in isolation but are embedded within broader developmental and sociodemographic contexts. Gender differences in adolescent mental health are well established, with girls showing higher levels of internalizing symptoms than boys [42–44]. Girls are also more likely to engage in rumination/negative thinking and use interpersonal ER strategies, whereas boys more often report greater use of avoidant or disengagement ER strategies (e.g., [4, 44]). These systematic subgroup differences suggest that regulatory configurations may vary not only in structure, but also in prevalence and psychological significance across gender groups. Accordingly, examining how strategies cluster within individuals may help capture developmentally and contextually sensitive patterns of emotion regulation and clarify their associations with mental health.

## The present study

The present study aims to identify ER profiles through latent profile analysis (LPA) in a large community sample of Swedish adolescents using six ER strategies assessed by the AERSQ–E: rumination/negative thinking, positive reorientation, social support, aggressive outlet, creative expression, and distraction [8, 20]. In addition, the study examines how the identified ER profiles are associated with mental health outcomes, including life satisfaction and symptoms of depression, anxiety, and NSSI, the latter of which is frequently linked to ER difficulties (e.g., [40]).

Based on previous research (e.g., [10, 14, 28]), it is hypothesized that profiles dominated by rumination/negative thinking will be associated with lower life satisfaction and higher levels of psychological problems. Conversely, adolescents with profiles characterized by positive reorientation are expected to report more favourable mental health outcomes, including higher life satisfaction and lower levels of anxiety, depression, and NSSI. However, more specific hypotheses (e.g., regarding the number of profiles or particular profile configurations) are constrained by the limited number of person-centred studies using ER strategy measures specifically developed for adolescents. As highlighted in Brandão’s [16] review, most strategy-focused studies have relied on instruments that primarily assess intrapersonal cognitive strategies, with comparatively little attention to interpersonal strategies such as social support, and even less attention to strategies such as creative expression. Because these strategies are rarely incorporated into profile-based frameworks, it remains unclear how they co-occur with other regulatory strategies within adolescents’ broader repertoires. Accordingly, the present study adopts an exploratory approach to identify potentially novel configurations of ER strategies and to examine how these patterns relate to both positive and negative mental health outcomes during adolescence.

## Method

### Participants

The data used in the present study were drawn from the longitudinal Deliberate Self-Harm, Emotion Regulation and Interpersonal Relations in Youth project (known as the SoL project) at Department of Psychology, Lund University, Sweden. The present study utilized a community sample of students in Grades 7–9 from seven regular schools in a municipality in southern Sweden (*N* = 759, *M*_*age*_ = 13.90, *SD*_*age*_ = 0.75). Approximately 1,000 students were invited to participate, of whom 808 participated (response rate = 80%). Of these, 42 participants were excluded due to substantial missing data on the ER measure, and an additional seven participants were excluded as multivariate outliers based on analyses of the ER variables, resulting in a final sample of 759 participants. Of the final sample, 48.6% identified as girls, 49.8% as boys, and 1.6% as other gender identities.

### Measures

#### Emotion regulation strategies

The Adolescents’ Emotion Regulation Strategies Questionnaire - Extended (AERSQ-E; [8, 20]) was used to assess ER strategies. The AERSQ–E consists of 23 items distributed across six subscales: rumination/negative thinking, positive reorientation, social support, aggressive outlet, creative expression, and distraction. All items begin with the phrase “When I experience difficult emotions…” and are followed by strategy-specific statements (e.g., rumination/negative thinking: “I think that I am bad or worthless”; positive reorientation: “I try to find something positive in what has happened”; social support: “I seek support and comfort from others”; aggressive outlet: “I try to find something to break”; creative expression: “I express my feelings by creating something”; distraction: “I try to think about something else”). Responses were recorded on a five-point Likert scale ranging from 0 (never) to 4 (very often/always). In the present study, the AERSQ–E demonstrated acceptable to strong internal consistency across subscales: rumination/negative thinking (α = 0.82), positive reorientation (α = 0.81), social support (α = 0.81), aggressive outlet (α = 0.81), creative expression (α = 0.71), and distraction (α = 0.65).

#### Life satisfaction

The Students’ Life Satisfaction Scale (SLSS; [45]) was used to assess perceived global life satisfaction. The scale consists of six items (e.g., “I have a good life”) rated on a four-point Likert scale ranging from 1 (never) to 4 (almost always). In the present study, the scale demonstrated strong internal consistency (α = 0.89).

#### Anxiety and depression

The Revised Children’s Anxiety and Depression Scale–25 (RCADS-25; [46, 47]) was used to assess symptoms of depression and anxiety. Ten items addressed depression (e.g., “I feel worthless”) and fifteen items assessed anxiety (e.g., “I worry what other people think of me”). Each item was rated on a Likert scale ranging from 0 (never) to 3 (always). In the present study, both the depression and anxiety subscales demonstrated strong internal consistency (α = 0.90).

#### Non–suicidal self–injury

The Deliberate Self–Harm Inventory (DSHI-9r; [48–50]) was used to assess NSSI during the past 6 months by asking adolescents whether they had intentionally engaged in any of nine different forms of self-injurious behaviour (e.g., “Have you intentionally hit yourself or banged your head against something so hard that it left a bruise?”). Each item was rated on a seven-point frequency scale, ranging from 0 (never) to 6 (more than five times). NSSI was considered repetitive when ≥ 5 instances of self-injury were reported. In the present study, the DSHI-9r demonstrated strong internal consistency (α = 0.91).

### Procedure

All regular schools within a municipality in southern Sweden, comprising approximately 40,000 residents, were invited to participate in the project, and all schools agreed to take part. Data were collected in 2023 as part of a larger research project. Ethical approval was obtained from the Swedish Ethical Review Authority (registration number 2021–06695–01) prior to the commencement of data collection.

Prior to data collection, written information about the study’s aims, procedures, voluntary nature, and confidentiality was distributed to adolescents and their parents or legal guardians. A passive parental consent procedure, approved by the ethical review authority, was employed, whereby parents were instructed to contact the research team if they did not consent to their child’s participation. Adolescents provided active informed consent before completing the survey and were clearly informed that participation was voluntary and that they could withdraw at any time without negative consequences. Data collection was conducted during regular school hours in a classroom setting. A trained research assistant administered the survey, and a teacher was present to help maintain a calm and orderly environment. Participants completed the questionnaire on their school laptops, and the survey took approximately 30 min to complete. To ensure participant safety and access to support, the school counsellor was informed in advance about the study and its content. Adolescents were also provided with written information on how to contact a clinical psychologist affiliated with the project should they experience distress or have questions following their participation.

### Data analysis

Preliminary data screening was conducted before the latent profile analyses (LPA). Participants with missing data on the majority of the AERSQ–E items were excluded. Multivariate outliers across the AERSQ–E subscales were identified using Mahalanobis distance and were also excluded. Item-level missingness was low (< 5%) for both the AERSQ–E and the distal outcome measures. Missing items were imputed using the Expectation–Maximization algorithm when participants had completed at least 80% of the items within a scale. Participants who completed fewer than 80% of the items received no score for that scale; consequently, the number of available observations varied slightly across the distal outcome analyses. Pearson correlations were used to examine associations among the ER strategies and mental health outcomes.

LPAs were conducted in Mplus Version 8 to identify profiles based on the six AERSQ–E strategies. Models specifying one to six profiles were estimated. Model selection was based on statistical fit, classification quality, profile size, interpretability, and parsimony. Statistical fit was evaluated using the Akaike Information Criterion (AIC), Bayesian Information Criterion (BIC), and sample-size-adjusted BIC (SABIC), with lower values indicating better fit. The Lo–Mendell–Rubin adjusted likelihood ratio test (LMR) and bootstrap likelihood ratio test (BLRT) were used to compare successive models; significant *p*-values indicated that a model with *k* profiles provided a better fit than a model with *k* − 1 profiles [51]. Entropy and average posterior probabilities were examined to assess classification quality [52]. Profile sizes were also inspected, with the criterion that no profile should contain fewer than 4% of the sample [53]. Models were estimated with equal within-profile variances across profiles and within-profile covariances fixed to zero.

Gender differences in profile membership were examined using the R3STEP procedure in Mplus, which accounts for classification uncertainty when estimating associations between covariates and profile membership [54]. Participants identifying with another gender identity were retained in the primary analyses but excluded from analyses comparing girls and boys because the group was too small to analyze separately.

Associations between the identified profiles and the distal outcomes (life satisfaction, anxiety, depression, and NSSI) were initially examined using participants’ most likely profile membership. The distributions of the outcomes and the homogeneity of variances across profiles were assessed before the group comparisons. Life satisfaction, anxiety, and depression were examined using one-way ANOVAs followed by Games–Howell post hoc tests. Because continuous DSHI-9r scores did not meet the parametric assumptions, profile differences in NSSI were examined using the Kruskal–Wallis test followed by Dwass–Steel–Critchlow–Fligner pairwise comparisons. Univariate outliers were retained because substantial variability was expected in this community sample.

Profile differences in the prevalence of repetitive NSSI, defined as five or more instances during the preceding six months, were examined using a chi-square test of independence. Significant omnibus differences were followed by pairwise *z*-tests of proportions with Bonferroni-adjusted *p*-values. Partial eta squared (*ηp²*) was reported for the ANOVAs, epsilon squared (*ε²*) for the Kruskal–Wallis test, and Cramér’s V for the chi-square test. Values of 0.01, 0.06, and 0.14 were interpreted as small, medium, and large effects, respectively, for *ηp²* and *ε²*. For Cramér’s V, values of 0.10, 0.30, and 0.50 were interpreted as small, medium, and large effects, respectively. The same outcome analyses were conducted for the supplementary six-profile solution.

To account for classification uncertainty, profile differences in life satisfaction, anxiety, depression, and continuous NSSI scores in the retained five-profile solution were additionally examined using the Bolck–Croon–Hagenaars (BCH) procedure in Mplus. Unlike analyses based solely on participants’ most likely profile membership, the BCH procedure incorporates posterior classification probabilities and thereby provides less biased estimates of differences in distal outcomes across latent profiles [54].

For the descriptive analyses of symptom severity, RCADS–25 raw scores were converted to T-scores using Swedish normative data [55]. Scores were categorized as low severity or non-clinical (T-score ≤ 64), medium severity or subclinical (T-score = 65–69), and high severity or clinical (T-score ≥ 70). Percentages were calculated among participants with available T-scores; consequently, denominators varied slightly across outcomes and profiles. For graphical presentation, continuous mental health outcomes were standardized as *z*-scores across the analytic sample.

## Results

### Correlational analyses

Initial analyses examined intercorrelations among the six ER strategies (see Table 1) and their associations with mental health outcomes (see Table 2). Correlations among ER strategies were weak to moderate, with the strongest association observed between positive reorientation and distraction for both girls (*r* = 0.58, *p* < 0.001) and boys (*r* = 0.51, *p* < 0.001), showing that this was the strongest strategy intercorrelation in both gender groups.

**Table 1:** Intercorrelations among emotion regulation strategies for girls (upper triangle) and boys (lower triangle), and means (SDs) for the total sample

Emotion regulation strategy	1	2	3	4	5	6
1. Rumination/negative thinking	-	− 0.44***	− 0.09	0.45***	0.12*	− 0.19***
2. Positive reorientation	− 0.02	-	0.34***	− 0.40***	0.13*	0.58***
3. Social support	0.29***	0.34***	-	− 0.02	0.20***	0.26***
4. Aggressive outlet	0.38***	− 0.16**	0.12*	-	0.11*	− 0.17***
5. Creative expression	0.39***	0.07	0.32***	0.27***	-	0.18***
6. Distraction	0.20***	0.51***	0.33***	0.05	0.07	-
Mean (SD)	1.45 (1.12)	0.95 (0.97)	1.77 (1.04)	0.97 (0.96)	0.52 (0.70)	1.97 (1.02)

Subscale scores for each emotion regulation strategy were calculated as the mean of the items comprising the subscale, yielding scores ranging from 0 (never) to 4 (almost always/always), with 2 (sometimes) representing the midpoint of the five-point response scale

To account for multiple testing, Bonferroni corrections were applied separately within each gender (15 correlations per gender; adjusted significance threshold: *p* < .0033 [0.05/15]); therefore, only correlations marked with *** are significant at this level

*p < 0.05; **p < 0.01; ***p < 0.001

**Table 2:** Pearson correlations between emotion regulation strategies and mental health outcomes for girls/boys

Emotion regulation strategy	Life satisfaction	Anxiety	Depression	NSSI
Rumination/negative thinking	− 0.60***/-0.43***	0.65***/0.59***	0.64***/0.48***	0.40***/0.37***
Positive reorientation	0.57***/0.36***	− 0.41***/-0.11*	− 0.52***/-0.28***	− 0.42***/-0.14**
Social support	0.27***/0.07	− 0.11*/0.23***	− 0.21***/0.00	− 0.15**/0.08
Aggressive outlet	− 0.48***/-0.29***	0.44***/0.22***	0.53***/0.29***	0.43***/0.26***
Creative expression	− 0.07/-0.16**	0.11*/0.28***	0.11*/0.17***	0.15**/0.36***
Distraction	0.27***/0.14**	− 0.16**/0.08	− 0.17**/0.04	− 0.20***/-0.04

*NSSI* Non-Suicidal Self Injury

To account for multiple testing, Bonferroni corrections were applied separately within each gender (16 correlations per gender; adjusted significance threshold: *p* < .0031 [0.05/16]); therefore, only correlations marked with *** are significant at this level

*p < .05; **p < .01; ***p < .001

As shown in Table 2, rumination/negative thinking was moderately to strongly related to mental health problems across genders, correlating positively with anxiety, depression, and NSSI (*r*s ≈ 0.37–0.65) and negatively with life satisfaction (*r*s ≈ –0.43–0.60). In contrast, positive reorientation was moderately positively related to life satisfaction (*r*s ≈ 0.36–0.57) and weakly to moderately negatively related to internalizing symptoms (*r*s ≈ –0.11–0.52), with generally stronger associations among girls. Social support showed small and gender-specific associations. Among girls, social support was positively related to life satisfaction (*r* = 0.27) and negatively related to anxiety (*r* = –0.11) and depression (*r* = –0.21), whereas among boys it was positively associated with anxiety (*r* = 0.23). Creative expression was weakly to moderately related to anxiety, depression, and NSSI (*r*s ≈ 0.10–0.35), particularly among boys (e.g., NSSI: *r* = 0.35), and showed weak or nonsignificant associations with life satisfaction. Aggressive outlet was consistently associated with mental health problems (*r*s ≈ 0.20–0.45), while distraction showed small positive associations with both symptoms and life satisfaction (*r*s ≈ 0.10–0.30), mainly among girls.

### Identifying emotion regulation profiles

Latent profile analyses were conducted to identify distinct ER profiles within the dataset. Models with one to six profiles were tested. As shown in Table 3, fit indices (AIC, BIC, and SABIC) decreased with additional profiles, with the lowest values observed for the six-profile solution. However, the statistical evidence for adding a sixth profile was mixed. Although the six-profile solution showed lower information criteria, the LMR test was not significant (*p* = .204), providing no statistical support from this test for adding a sixth profile relative to the five-profile solution. Both solutions showed good and comparable classification quality. The five-profile solution had an entropy of 0.81 and average posterior probabilities ranging from 0.86 to 0.89, whereas the corresponding values for the six-profile solution were 0.80 and 0.81–0.89, respectively. For both solutions, the best log-likelihood value was replicated across random starts, providing additional evidence that the solutions were consistently reproduced.

**Table 3:** Latent profile analysis model fit summary

Model	Log likelihood	AIC	BIC	SABIC	Entropy	LMR *p*-value	BLRT *p*-value	Proportions
1	-6261.20	12546.39	12601.97	12563.87	1.00			1.00
2	− 6030.27	12098.54	12186.55	12126.21	0.90	< 0.001	< 0.001	0.80, 0.20
3	− 5891.09	11834.19	11954.62	11872.06	0.82	0.836	< 0.001	0.62, 0.20, 0.18
4	− 5781.82	11629.64	11782.50	11677.71	0.79	0.005	< 0.001	0.16, 0.44, 0.24, 0.16
**5**	**− 5703.96**	**11487.92**	**11673.20**	**11546.19**	**0.81**	**0.024**	**< 0.001**	**0.25**,** 0.16**,** 0.13**,** 0.07**,** 0.39**
6	− 5643.380	11380.76	11598.44	11449.22	0.80	0.204	< 0.001	0.14, 0.06, 0.24, 0.36, 0.11, 0.08

Selected model is highlighted in bold. *AIC* Akaike Information Criterion, *BIC* Bayesian Information Criterion, *SABIC* Sample-size adjusted BIC, *LMR* Lo-Mendell-Rubin likelihood ratio test, *BLRT* Bootstrap Likelihood Ratio Test

Inspection of the profile configurations showed that the six-profile solution largely reproduced the overall structure of the five-profile solution but further differentiated the Creative Rumination-Aggression profile into two more specific configurations (see Figure S1, Supplementary Materials). One was characterized by elevated rumination/negative thinking, aggressive outlet, and creative expression, whereas the other was distinguished by particularly elevated aggressive outlet without a corresponding elevation in creative expression and with comparatively lower rumination/negative thinking. Thus, the six-profile solution identified an additional Aggressive Outlet–Focused profile while otherwise largely preserving the profile structure observed in the five-profile solution.

Given the nonsignificant LMR test, comparable classification quality, largely similar overall profile structure, and greater parsimony, the five-profile solution was retained as the primary solution. However, the six-profile solution provided potentially meaningful additional differentiation within the Creative Rumination-Aggression profile and was therefore regarded as a viable alternative. The six-profile configuration and corresponding analyses of the mental health outcomes are presented in the Supplementary Materials (Figure S1 and Table S1).

### Profile characterization

**Fig. 1:**
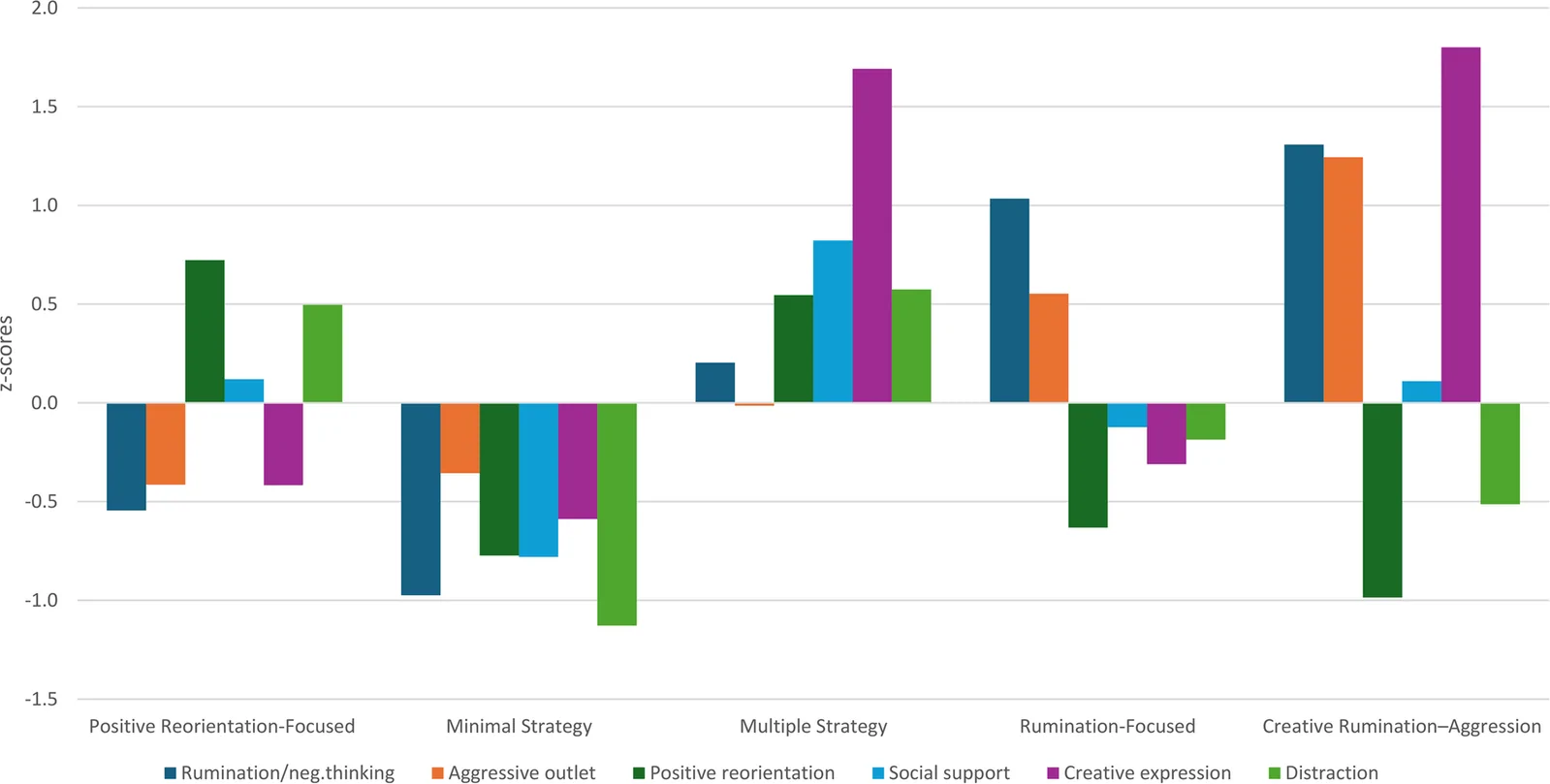
Z scores for emotion regulation strategies across five emotion regulation profiles

After retaining the five-profile solution as the primary and more parsimonious solution, each profile was conceptually interpreted and labelled according to its distinctive pattern of ER strategy use (see Fig. 1). The Positive Reorientation–Focused profile (*n* = 296; 39%) was characterized by high use of positive reorientation and distraction together with notably low rumination/negative thinking. The Minimal Strategy profile (*n* = 125; 16.5%) showed generally low engagement across all ER strategies, with most scores falling more than 0.5 *SD* below the sample mean. The Multiple Strategy profile (*n* = 97; 12.8%) was characterized by broad use of strategies, including particularly high creative expression and above-average use of positive reorientation, social support, and distraction. The Rumination–Focused profile (*n* = 190; 25%) was distinguished by high levels of rumination/negative thinking (approximately 1 *SD* above the mean) and above-average aggressive outlet, combined with comparatively low use of positive reorientation. Finally, the Creative Rumination-Aggression profile (*n* = 51; 6.7%) showed markedly elevated use of aggressive outlet, rumination/negative thinking, and creative expression (1.24–1.80 *SD* above the mean), along with below-average levels of positive reorientation and distraction. Table 4 shows significant differences in strategy use across profiles (*p* < 0.001 for all overall tests), with large effect sizes (*ηp²*s = 0.20–0.73), indicating substantial variability in ER strategy use across the profiles.

**Table 4:** Means (SDs) and ANOVA results for differences across emotion regulation profiles

Variable	Emotion Regulation Profile					F	η^2^_*p*_
	Positive Reorientation-Focused *n* = 296, 39.0%	Minimal Strategy *n* = 125, 16.5%	Multiple Strategy *n* = 97, 12.8%	Rumination-Focused *n* = 190, 25.0%	Creative Rumination–Aggression *n* = 51, 6.7%		
Rumination/negative thinking	0.84 (0.65)	0.36 (0.46)	1.67 (0.75)	2.60 (0.69)	2.91 (0.71)	367.04	0.66
Positive reorientation	2.52 (0.72)	0.96 (0.70)	2.33 (0.84)	1.11 (0.75)	0.74 (0.60)	192.87	0.51
Social support	1.08 (0.94)	0.22 (0.48)	1.76 (1.01)	0.85 (0.83)	1.07 (0.52)	46.26	0.20
Aggressive outlet	0.55 (0.62)	0.60 (0.86)	0.93 (0.78)	1.49 (1.07)	2.16 (0.89)	69.18	0.27
Creative expression	0.23 (0.33)	0.10 (0.25)	1.70 (0.48)	0.30 (0.35)	1.77 (0.52)	517.75	0.73
Distraction	2.48 (0.76)	0.81 (0.77)	2.56 (0.78)	1.78 (0.92)	1.44 (0.84)	112.70	0.37

*η*_*p*_² Partial eta squared

To account for multiple testing, a Bonferroni correction was applied across the six omnibus ANOVAs (adjusted significance threshold: *p* < .0083 [0.05/6]). All omnibus tests remained significant following this correction (all *p*s < 0.001). Pairwise comparisons were conducted using the Games–Howell post hoc test

Gender composition differed significantly across the latent profiles. The Positive Reorientation–Focused profile and the Minimal Strategy profile included predominantly boys (66% and 77%, respectively), whereas the Multiple Strategy profile, the Rumination–Focused profile, and the Creative Rumination–Aggression profile included predominantly girls (66.7%, 72.3%, and 87.8%, respectively). R3STEP analyses confirmed this pattern, showing significant differences in gender composition between most profile pairs. The only non-significant comparisons were between Profiles 1 and 2 (34% vs. 23% girls, *p* = 0.057), Profiles 3 and 4 (67% vs. 72% girls, *p* = 0.079), and Profiles 4 and 5 (72% vs. 88% girls, *p* = 0.147).

### Mental health differences across emotion regulation profiles

Significant differences were observed across the five profiles for all mental health outcomes (all *p*s < 0.001; Table 5), with large effect sizes across the different effect-size measures. Post hoc comparisons revealed significant differences between most profile pairs. To account for classification uncertainty, the distal outcome analyses were repeated using the BCH procedure in Mplus. The BCH analyses confirmed significant overall differences across the five profiles in life satisfaction, anxiety, depression, and NSSI (all χ²s = 154.16–358.20, all *p*s < 0.001).

**Table 5:** Means (standard deviations), prevalence, and omnibus test results for mental health outcomes across the five emotion regulation profiles

Variable	Emotion Regulation Profile					Omnibus test	Effect size
	Positive Reorientation-Focused *n* = 296, 39.0%	Minimal Strategy *n* = 125, 16.5%	Multiple Strategy *n* = 97, 12.8%	Rumination-Focused *n* = 190, 25.0%	Creative Rumination–Aggression *n* = 51, 6.7%		
Life satisfaction	18.25(4.12)	16.67 (5.03)	15.85 (4.47)	11.26 (4.97)	8.67 (5.06)	93.72	0.34
Anxiety	8.01 (6.39)	6.96 (5.40)	12.89 (7.76)	18.01 (8.35)	23.39 (8.36)	100.26	0.36
Depression	5.85 (3.83)	6.43 (4.69)	9.10 (5.34)	13.70 (6.40)	17.34 (6.94)	102.91	0.37
NSSI, total score	1.12 (3.51)	2.38 (6.81)	6.17 (10.28)	10.74 (14.14)	21.71 (18.47)	171.47	0.24
NSSI repetitive	9.6%	17.9%	35.2%	49.7%	75.5%	150.32	0.46

*NSSI* Non-suicidal self-injury

Values are means (standard deviations), except for repetitive NSSI, which is presented as the percentage within each profile reporting ≥ 5 instances. Life satisfaction, anxiety, and depression were examined using one-way ANOVAs, continuous NSSI scores using the Kruskal–Wallis test, and repetitive NSSI using a chi-square test. Partial eta squared (*ηp²*) is reported for the ANOVAs, epsilon squared (*ε²*) for the Kruskal–Wallis test, and Cramér’s V for the chi-square test. A Bonferroni correction was applied across the five omnibus tests; adjusted significance threshold was *p* < .010 (.05/5)

Distal outcomes were additionally examined using the BCH procedure in Mplus to account for classification uncertainty. These analyses confirmed significant overall profile differences in life satisfaction, anxiety, depression, and NSSI (all *p*s < 0.001)

Because the latent profiles also differed significantly in gender composition, additional analyses were conducted adjusting for gender. The overall pattern of findings remained unchanged. After adjustment for gender, the profiles continued to differ significantly in life satisfaction, anxiety, depression, and continuous NSSI total scores (all *F*s = 38.48–71.45, all *p*s < 0.001, *ηp²*s = 0.18–0.29). Although gender was independently associated with each outcome (all *p*s < 0.001), the profile differences remained significant after adjustment for gender.

**Fig. 2:**
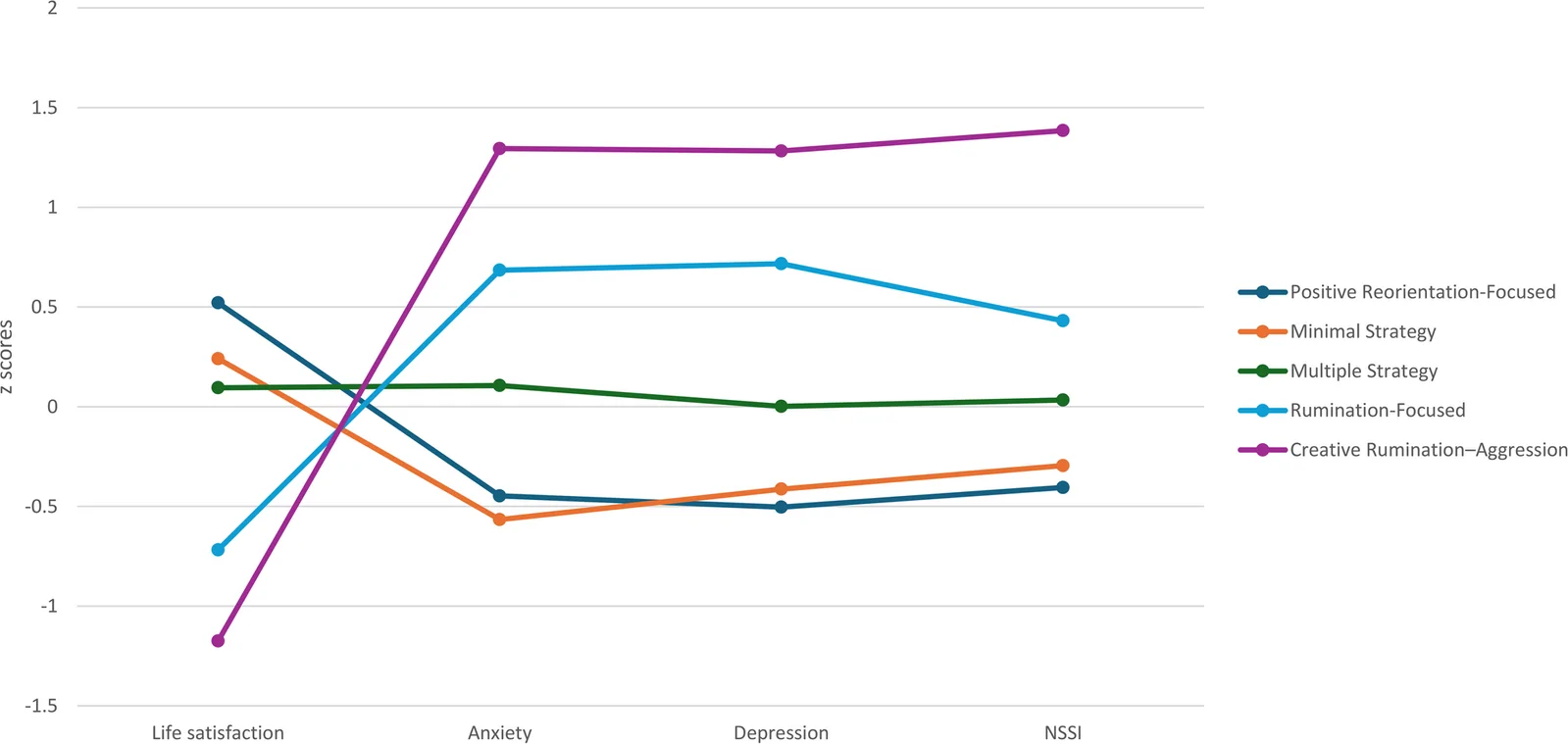
Mental health outcomes (Z scores) across five emotion regulation profiles

Figure 2 illustrates the mental health patterns across the ER profiles. The Positive Reorientation–Focused and Minimal Strategy profiles showed the most favourable mental health, with higher life satisfaction (≈ 1 *SD* above the mean) and lower levels of anxiety and depression (≈ 0.5–1 *SD* below the mean). In contrast, the Rumination–Focused and the Creative Rumination-Aggression profiles exhibited the highest levels of mental health problems, with life satisfaction approximately 1.5–2 *SD*s below the mean and anxiety and depression approximately 1–2 *SD*s above the mean. The Multiple Strategy profile scored close to the sample mean (± 0.3 *SD* across variables), reflecting intermediate levels of mental health. Consistent with these findings, NSSI levels were highest in the Creative Rumination-Aggression profile and elevated in the Rumination–Focused profile, whereas the Positive Reorientation–Focused profile showed the lowest levels (see Table 5).

### Variations in symptom severity across profiles

**Fig. 3:**
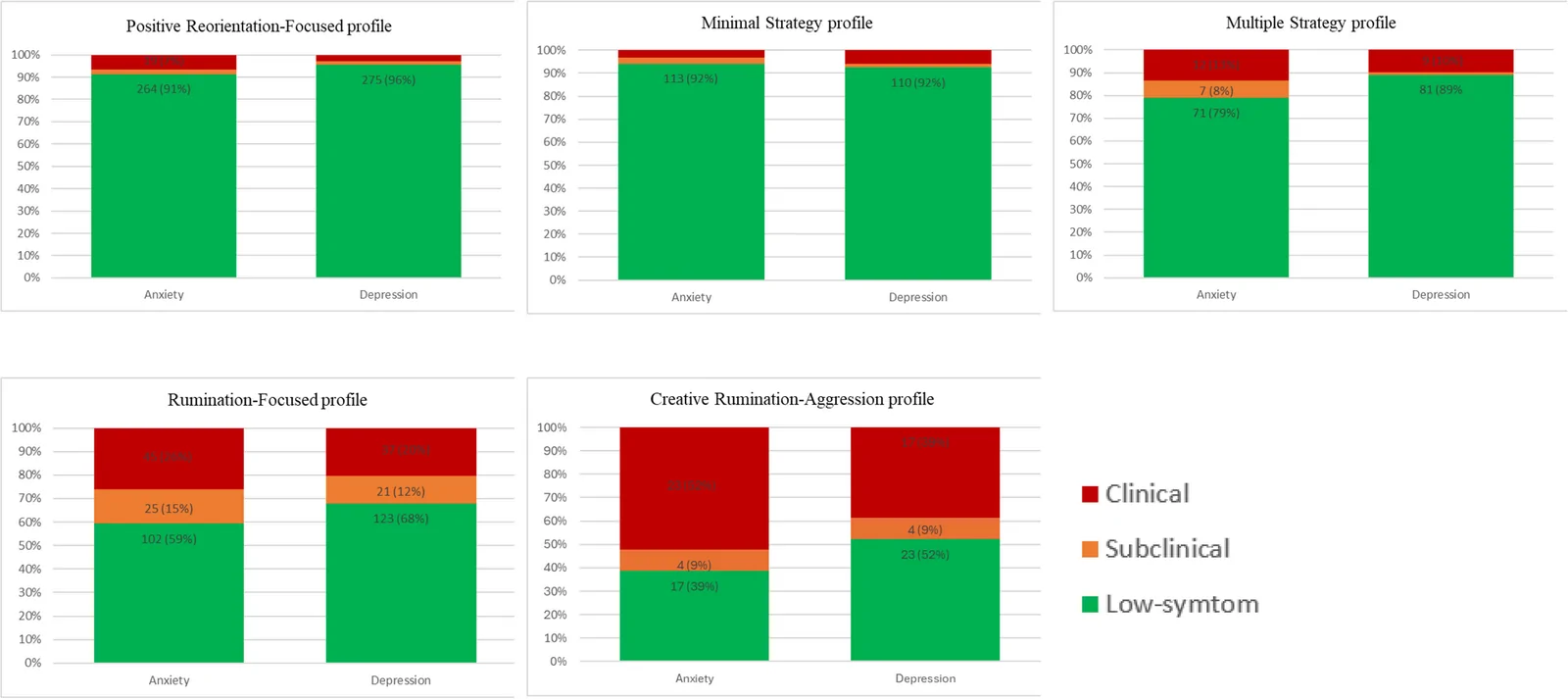
Distribution of clinical, subclinical, and low-symptom levels of anxiety and depression across the five emotion regulation profiles

To further explore differences in symptom severity, participants’ RCADS–25 raw scores were converted to T-scores and categorized into non-clinical, subclinical, and clinical levels of anxiety and depression. In the total sample, 10.5% (*n* = 78) scored within the clinical range for depression and 13.9% (*n* = 103) for anxiety. These proportions varied across profiles, as shown in Fig. 3. The Positive Reorientation–Focused and Minimal Strategy profiles had the lowest proportions of adolescents in the clinical range. Specifically, only 2.7% (*n* = 8) and 5.8% (*n* = 7) of adolescents in these profiles, respectively, scored in the clinical range for depression, and 6.5% (*n* = 19) and 3.3% (*n* = 4) scored in the clinical range for anxiety. The Multiple Strategy profile showed rates similar to those of the total sample, with 9.6% (*n* = 9) scoring in the clinical range for depression and 12.8% (*n* = 12) for anxiety. In contrast, the Rumination–Focused and Creative Rumination-Aggression profiles contained the highest proportions of adolescents scoring in the clinical range for both depression and anxiety. In the Rumination–Focused profile, 19.8% (*n* = 37) scored in the clinical range for depression and 24.1% (*n* = 45) for anxiety. The Creative Rumination-Aggression profile showed the most severe pattern, with 34.7% (*n* = 17) of adolescents in the clinical range for depression and 48.9% (*n* = 23) for anxiety.

## Discussion

The present study identified five distinct ER strategy profiles among Swedish adolescents based on the AERSQ–E [20]. The profiles were labelled according to their predominant ER strategies and are presented here in order of increasingly unfavourable mental health outcomes: Positive Reorientation–Focused, Minimal Strategy, Multiple Strategy, Rumination–Focused, and Creative Rumination-Aggression. The profiles showed substantial differences in their patterns of ER strategy use and also differed significantly across all mental health outcomes, with large effect sizes. These findings indicate substantial heterogeneity in adolescents’ ER and suggest that distinct constellations of ER strategies are systematically associated with differences in mental health. Three findings are particularly noteworthy. First, three ER profiles were associated with comparatively favourable mental health outcomes, suggesting that favourable mental health may be associated with multiple regulatory configurations. Notably, the Minimal Strategy profile was characterized by limited strategy use and, contrary to prior findings, was nonetheless associated with favourable mental health outcomes. Second, consistent with prior research, profiles characterized by high rumination/negative thinking were associated with elevated anxiety, depression, and NSSI. The Creative Rumination-Aggression profile showed the most severe pattern, with nearly half of the adolescents scoring within the clinical range for anxiety, indicating that this profile was concurrently associated with particularly high levels of psychological difficulties. Third, creative expression appeared in both profiles associated with more favourable mental health and those associated with greater psychological difficulties, suggesting that the associations of specific strategies with mental health may differ depending on the broader configuration in which they co-occur. Accordingly, the mental health correlates of a strategy may be better understood by considering the broader regulatory pattern in which it occurs rather than examining the strategy in isolation.

### Profiles associated with favourable mental health

The Positive Reorientation–Focused and Minimal Strategy profiles were characterized by the most favourable mental health, whereas the Multiple Strategy profile showed generally intermediate outcomes. Nevertheless, all three profiles showed more favourable outcomes than the Rumination–Focused and Creative Rumination-Aggression profiles. This pattern suggests that favourable mental health during adolescence may be associated with multiple regulatory patterns rather than a single optimal configuration. Two of these profiles, the Positive Reorientation–Focused and Multiple Strategy profiles, shared elevated levels of positive reorientation, a strategy consistently linked to lower internalizing symptoms and favourable mental health in variable-oriented research [2, 41]. The prominence of positive reorientation in these profiles is consistent with its previously demonstrated associations with favourable emotional functioning in adolescence. Both profiles also demonstrated elevated use of distraction, which in the present study was positively associated with favourable mental health. Distraction was moderately correlated with positive reorientation, indicating that these strategies tended to co-occur. From a process model perspective [24], distraction reflects attentional deployment and may reduce emotional intensity in the short term by shifting focus away from distressing stimuli. Such a shift could potentially create cognitive space for subsequent reappraisal or reframing. However, when distraction occurs in the absence of cognitive restructuring, it may resemble avoidance, which has been linked to higher levels of psychological problems [2]. The present findings show that distraction formed part of profiles associated with favourable mental health when it co-occurred with positive reorientation. This pattern is consistent with the possibility that the mental health correlates of distraction may differ depending on the broader configuration in which it occurs. However, the present findings do not establish how distraction operates within these profiles. The favourable outcomes observed in the Multiple Strategy profile are consistent with prior person-centred research showing that broader strategy repertoires are associated with better psychological adjustment [14]. Similarly, theoretical accounts emphasize that effective ER involves adapting strategies to contextual demands rather than relying rigidly on a narrow set of responses [20, 56]. However, broader strategy use should not itself be interpreted as evidence of regulatory flexibility, which involves adapting strategy use to contextual demands and was not directly assessed in the present study.

The comparatively favourable mental health observed in the Minimal Strategy profile warrants particular consideration. Low-engagement profiles have been identified in previous person-centred studies [10, 14], yet their associations with adjustment have been inconsistent. For example, Lougheed and Hollenstein [14] linked limited strategy use to higher internalizing symptoms relative to profiles characterized by broader strategy use, whereas Otterpohl et al. [10] found that the poorest outcomes were observed in profiles characterized by elevated maladaptive strategies rather than minimal strategy use. These findings raise the possibility that poorer mental health may be more closely associated with the presence of less adaptive strategies than with low overall strategy use per se. Consistent with this interpretation, minimal strategy use in the present study was not associated with poorer mental health outcomes, as reflected in levels of anxiety, depression, and NSSI. Indeed, adolescents in this profile reported mental health that was nearly as favourable as that in the Positive Reorientation–Focused profile. One possible explanation is that adolescents in this profile may experience lower emotional intensity or fewer stressors, thereby reducing the need for active regulatory efforts. This interpretation aligns with contextual and flexibility-based models of ER, which emphasize that effectiveness depends on situational demands rather than frequent strategy use [56, 57]. Alternatively, minimal reported engagement may reflect relatively automatic or implicit regulatory processes that are not fully captured by self-report measures [58]. Differences across studies may also stem from variation in measurement instruments, developmental stages, sample characteristics, or the specific emotional domains assessed. These factors may influence how minimal strategy use is captured and interpreted across studies. Further research is therefore needed to clarify the nature of the Minimal Strategy profile and its implications for mental health. In particular, longitudinal research is required to determine whether its comparatively favourable concurrent mental health pattern remains stable over time and how it relates to regulatory demands.

### Profiles associated with poorer mental health

Profiles characterized by elevated rumination/negative thinking, particularly when combined with aggressive outlet, were associated with the most pronounced mental health difficulties. Both the Rumination–Focused profile and the Creative Rumination-Aggression profile displayed substantially higher levels of anxiety, depression, and NSSI than the other profiles. Notably, the Creative Rumination-Aggression profile exhibited the most severe pattern, with nearly half of adolescents scoring within the clinical range for anxiety and 75.5% reporting repetitive NSSI. From a variable-oriented perspective, these findings are consistent with prior research linking rumination/negative thinking to internalizing symptoms and NSSI [6, 8, 30, 31], and aggressive outlet to emotional and behavioural dysregulation [59]. However, the present person-centred results extend this work by showing that the co-occurrence of these strategies within the same regulatory configuration was associated with particularly high levels of psychological difficulties. Thus, the findings identify a subgroup in which rumination/negative thinking and aggressive outlet co-occur with particularly poor mental health. However, they do not establish whether this combination of strategies contributes to psychological difficulties, emerges in response to such difficulties, or reflects reciprocal associations. One possible theoretical interpretation of this pattern is provided by the functional emotion regulation model of NSSI [37–40], which proposes that self-injury may serve as a maladaptive means of regulating overwhelming negative affect when more adaptive ER strategies are ineffective or unavailable. From this perspective, the observed combination of high rumination/negative thinking and aggressive outlet could reflect difficulties in emotion regulation. However, neither the emotion-regulatory function of NSSI nor the processes linking these strategies to NSSI were directly assessed in the present study.

The supplementary six-profile solution provided a further refinement of the Creative Rumination-Aggression profile. Specifically, the Creative Rumination-Aggression profile from the five-profile configuration was divided into two more specific profiles. In the six-profile solution, Cluster 5 retained the elevated creative expression characteristics of the original profile, together with relatively elevated rumination/negative thinking and aggressive outlet. In contrast, Cluster 6, labelled the Aggressive Outlet-Focused profile, was characterized primarily by elevated aggressive outlet, without elevated creative expression and with lower levels of rumination/negative thinking. This distinction suggests that the broader Creative Rumination-Aggression profile identified in the five-profile solution may encompass more than one configuration of ER strategy use.

The six-profile solution also showed significant overall differences across all mental health outcomes, although the two additionally differentiated profiles showed broadly similar concurrent mental health patterns. These validation findings are considered complementary to the structural distinction between the profiles rather than evidence for selecting one profile solution over the other. Longitudinal research and replication in independent samples are needed to determine whether this additional differentiation represents a stable and developmentally meaningful ER configuration.

More broadly, the co-occurrence of rumination/negative thinking and aggressive outlet across these configurations may have several possible interpretations. One theoretically plausible explanation is that this combination reflects difficulties in effectively downregulating emotional arousal. Rumination/negative thinking is consistently associated with prolonged negative affect and impaired emotional recovery [2, 6]. Aggressive outlet, in contrast, may temporarily discharge arousal but does not promote cognitive reappraisal or emotional resolution [59, 60]. It is therefore possible that their co-occurrence reflects a regulatory context in which emotional activation is maintained rather than effectively resolved. However, this process was not directly examined in the present study. Importantly, the profiles associated with poorer mental health were also characterized by comparatively low use of certain strategies. Both the Rumination–Focused and Creative Rumination-Aggression profiles were characterized by comparatively low use of distraction and positive reorientation. As distraction involves shifting attention away from distressing stimuli [24, 60], limited use of this strategy may theoretically provide fewer opportunities to disengage from negative thought patterns, allowing rumination/negative thinking to persist [1]. Similarly, low positive reorientation indicates less frequent reported engagement in cognitive reframing processes linked to more favourable mental health [2, 41]. Consistent with the broaden–and–build model [61], lower engagement in such positive reinterpretation may theoretically be associated with less flexible cognitive processing and a more narrow, self-focused processing style. From a regulatory flexibility perspective [56, 57], this pattern may be consistent with more limited variation in reported strategy use. However, the present study did not assess whether adolescents were able to flexibly adapt their strategies to different contextual demands. Thus, poorer mental health may be associated not only with higher reported use of rumination/negative thinking and aggression, but also with lower engagement in potentially adaptive strategies. However, the present data cannot establish whether lower use of these strategies contributes to poorer mental health or instead reflects adolescents’ responses to greater psychological distress.

The prominence of girls within these profiles aligns with evidence that adolescent girls report higher levels of rumination/negative thinking, internalizing symptoms and negative emotionality than boys [42–44, 62–66]. Consistent with broader research on ER, adolescent girls also tend to rely more on repetitive negative thinking and other internalizing forms of emotion regulation [44, 62, 63]. Together, the findings suggest that the likelihood of membership in particular ER configurations differ by gender. At the same time, the predominance of boys in the Minimal Strategy profile suggests that comparatively favourable adjustment may also co-occur with lower overall engagement in emotion regulation, further illustrating that favourable mental health can co-occur with different patterns of strategy use. One possible explanation is that boys report lower use of emotion regulation strategies because they tend to be less aware of, or less likely to reflect on, their internal experiences [67]. However, this interpretation remains speculative. Importantly, these findings should not be interpreted as indicating that girls or boys follow uniform regulatory patterns, but rather as reflecting differences in the probability of membership in specific emotion regulation profiles.

Although the present findings should not be interpreted as establishing clinical risk profiles, they may have implications for prevention. The predominance of girls in the profiles associated with poorer mental health suggests that prevention and intervention efforts targeting rumination/negative thinking and strengthening adaptive emotion regulation strategies may be particularly relevant during adolescence. More broadly, adolescents characterized by higher levels of repetitive negative thinking combined with lower use of potentially adaptive emotion regulation strategies represented a subgroup with greater concurrent psychological difficulties in the present sample. If replicated in longitudinal and clinical samples, these findings could inform school-based prevention efforts and emotion regulation interventions aimed at strengthening more adaptive emotion regulation strategies while reducing reliance on strategies associated with poorer mental health [68]. However, given the cross-sectional design and community-based sample, these implications should be regarded as tentative and require further investigation. Longitudinal research is also needed to clarify the developmental mechanisms underlying these observed gender differences.

### Creative expression across profiles

Profiles associated with different levels of mental health were distinguished not only by the presence of specific strategies (e.g., positive reorientation or rumination/negative thinking), but also by how these strategies were configured within broader regulatory repertoires. Creative expression illustrates this configurational principle particularly well, as it appeared in profiles associated with both more favourable and poorer mental health.

Within the Creative Rumination-Aggression profile, creative expression co-occurred with elevated rumination/negative thinking and aggressive outlet, as well as relatively low levels of distraction and positive reorientation. In this context, it was associated with greater psychological difficulties. By contrast, creative expression also appeared in the Multiple Strategy profile, where it co-occurred with positive reorientation, distraction, and social support, and was not linked to poorer mental health. This contrast illustrates that the same strategy can occur within profiles characterized by substantially different levels of mental health. This pattern is consistent with the possibility that the mental health correlates of individual strategies may differ depending on the broader regulatory configuration in which they occur. This interpretation is also broadly consistent with regulatory flexibility frameworks, which emphasize that the adaptiveness of ER strategies depends on contextual fit and the availability of alternative regulatory options [56, 57]. However, regulatory flexibility itself was not directly assessed in the present study.

One possible theoretical interpretation is that creative expression may serve qualitatively different regulatory functions depending on how it is integrated into the broader regulatory pattern. Expressive activities such as writing, music, or art can facilitate emotional processing, cognitive reappraisal, and meaning-making, and have been associated with favourable mental health when they promote reflection, integration, and resolution [69, 70]. In this sense, creative expression could potentially complement strategies such as positive reorientation by helping adolescents transform distress into coherent narratives or future-oriented perspectives. However, when expressive engagement becomes repetitive and self-focused, it may overlap with perseverative processes such as rumination/negative thinking [1]. In profiles already characterized by high rumination/negative thinking and limited attentional shifting or reframing, creative expression could therefore potentially serve a different regulatory function when it co-occurs with these strategies than when it co-occurs with other strategies. These functional interpretations remain speculative, however, because the present study did not directly examine how creative expression operates within different regulatory configurations.

### Strengths, limitations, and future directions

The present study has several notable strengths. It draws on a large, community-based sample of adolescents, enhancing statistical power, reliability, and generalizability within this developmental period. Moreover, the use of a measure specifically developed for adolescents allowed the assessment of a broader range of ER strategies, including interpersonal and expressive strategies that are often underrepresented in other instruments. By applying a person-centred approach, the study captures naturally occurring constellations of co-occurring strategies, thereby offering a more nuanced understanding of ER patterns in adolescence, where strategies may co-occur within broader regulatory configurations.

At the same time, several limitations should be considered when interpreting the findings. First, baseline levels of emotional intensity were not assessed. Consequently, it remains unclear whether differences in strategy profiles reflect variation in regulatory tendencies or differences in exposure to emotionally challenging experiences. Adolescents who report minimal strategy use may experience lower emotional intensity and therefore have less need to regulate, whereas those exposed to more frequent or intense negative affect may report greater use of regulatory strategies. Future research should incorporate measures of emotional reactivity or stress exposure to better disentangle patterns of strategy use from regulatory demands. Second, emotional awareness and the ability to accurately identify one’s own strategy use were not directly assessed, which may have influenced the precision of reported profiles. Future studies would benefit from multi-method approaches, such as behavioural assessments, ecological momentary assessment, or informant reports. Third, the cross-sectional design precludes conclusions about directionality or developmental change. Although specific ER profiles were associated with more favourable or poorer mental health, causal relationships cannot be inferred. Moreover, because latent profile analysis assigns individuals to profiles probabilistically, the identified profiles should be viewed as the most likely emotion regulation patterns in the present sample rather than fixed categories. Additionally, as emotion regulation flexibility was not measured, it is not possible to determine whether membership in a profile characterized by the use of multiple strategies reflects regulatory flexibility. Similarly, interpretations concerning protective or vulnerability processes, or the functional interplay between strategies within profiles, should be regarded as theoretically informed explanations of the observed concurrent associations rather than mechanisms demonstrated by the present study.

Longitudinal research is needed to examine the structural and individual stability of the identified profiles and their prospective associations with mental health and NSSI. Importantly, both the five- and six-profile solutions should be evaluated longitudinally to determine which structure shows greater stability and whether the additional differentiation in the six-profile solution is developmentally meaningful. Replication in independent samples, age groups, and cultural contexts is also needed to establish the robustness and generalizability of the identified patterns. Such research could further clarify whether profiles associated with poorer concurrent mental health are prospectively associated with subsequent psychological difficulties. It should also be noted that the profiles identified in the present study are not definitive and require further investigation. Other patterns of emotion regulation strategies may emerge in different samples or when using different measures. Additional research is therefore needed to further explore the interplay among different emotion regulation strategies, other aspects of emotional functioning, and mental health outcomes. Finally, intervention-oriented studies could examine whether changes in strategies associated with more favourable mental health are accompanied by changes in broader regulatory patterns and psychological functioning, thereby informing preventive and clinical efforts.

## Conclusion

Using a person-centred approach, this study identified five distinct ER profiles among adolescents. The Positive Reorientation–Focused and Minimal Strategy profiles showed the most favourable mental health, whereas the Multiple Strategy profile showed intermediate outcomes. The Rumination-Focus and Creative Rumination-Aggression profiles showed the poorest mental health. The Creative Rumination-Aggression profile, which consisted predominantly of girls, showed the most severe concurrent mental health pattern, with nearly half of its members scoring within the clinical range for anxiety and 75.5% reporting repetitive NSSI. These findings underscore the heterogeneity of ER in adolescence and show that distinct patterns of ER are differentially associated with mental health. Notably, limited strategy use was not associated with poorer mental health in the present sample, and the associations between specific strategies, such as creative expression, and mental health differed across the broader regulatory configurations in which they co-occurred. By incorporating a broader range of strategies within a person-centred framework, the study highlights the value of examining naturally occurring constellations of emotion regulation strategies rather than isolated strategies. Longitudinal research is needed to determine the direction of these associations, examine the stability of both the five- and six-profile solutions, and clarify whether particular ER configurations are prospectively associated with subsequent mental health outcomes.

## Supplementary Information

Supplementary Material 1.

## Data Availability

The datasets generated and/or analysed during the current study are not publicly available due to ethical restrictions but are available from the corresponding author on reasonable request.
