# Supplementary material for: Mapping emotion regulation profiles in adolescence and examining their associations with mental health outcomes
Source: Child Adolesc Psychiatry Ment Health. 2026 Sep 25;20:134. doi: 10.1186/s13034-026-01180-8 (PMC13615630; doi:10.1186/s13034-026-01180-8)
Supplement: Supplementary file 1 [file 13034_2026_1180_MOESM1_ESM.docx]

**Supplementary Material**

**Figure S1**

*Z Scores for Emotion Regulation Strategies Across Six Emotion Regulation Profiles*

|  | Rumination/neg.thinking | Aggressive outlet | Positive reorientation | Social support | Creative expression | Distraction |
| --- | --- | --- | --- | --- | --- | --- |
| Positive Reorientation-Focused | -0.592406 | -0.472735 | 0.764646 | 0.116709 | -0.458572 | 0.496304 |
| Minimal Strategy | -0.995556 | -0.577966 | -0.762961 | -0.779133 | -0.625618 | -1.18207 |
| Multiple Strategy | 0.0666012 | -0.243796 | 0.647057 | 0.794715 | 1.64577 | 0.61482 |
| Rumination-Focused | 0.930246 | 0.141272 | -0.516822 | -0.120276 | -0.220474 | -0.10837 |
| Creative Rumination–Aggression | 1.11836 | 1.25142 | -0.648817 | 0.306497 | 1.89993 | -0.325086 |
| Aggressive Outlet-Focused | 0.621338 | 2.32169 | -0.98077 | -0.1445 | -0.329497 | -0.365685 |

**Table S1**

*Means (Standard Deviations), Prevalence, and Omnibus Test Results for Mental Health Outcomes Across the Six Emotion Regulation Profiles*

| Variable | Emotion Regulation Profile | | | | | |  | Omnibus test | Effect size |
| --- | --- | --- | --- | --- | --- | --- | --- | --- | --- |
|  | Positive Reorientation-Focused  n=276 | Minimal Strategy  n=110 | Multiple Strategy  n=81 | Rumination-Focused  n=182 | Creative Rumination-Aggression  n=61 | Aggressive Outlet-Focused  n=48 |  |  |  |
| Life satisfaction | 18.54  (4.00) | 17.07  (4.67) | 16.28  (4.21) | 12.05 (4.87) | 9.91  (5.42) | 9.90 (5.73) |  | 77.82 | .35 |
| Anxiety | 7.70  (6.18) | 6.95  (5.63) | 12.04  (7.83) | 16.82 (7.87) | 20.86 (8.32) | 18.59 (11.39) |  | 67.56 | .32 |
| Depression | 5.64  (3.73) | 6.05  (4.53) | 8.78  (5.23) | 12.73 (5.91) | 15.82 (7.12) | 14.91 (7.76) |  | 77.56 | .35 |
| NSSI, total score | 0.82  (2.44) | 1.98  (6.70) | 4.42  (7.81) | 9.23 (12.73) | 20.11 (17.24) | 14.14 (18.16) |  | 181.84 | .25 |
| NSSI repetitive | 7.3% | 14.7% | 31.1% | 45.8% | 73.7% | 59.1% |  | 170.02 | .49 |

*Note.* *NSSI* = non-suicidal self-injury.

Values are means (standard deviations), except for repetitive NSSI, which is presented as the percentage within each profile reporting ≥ 5 instances. Life satisfaction, anxiety, and depression were examined using one-way ANOVAs, continuous NSSI scores using the Kruskal–Wallis test, and repetitive NSSI using a chi-square test. Partial eta squared (*ηp²*) is reported for the ANOVAs, epsilon squared (*ε²*) for the Kruskal–Wallis test, and Cramér’s V for the chi-square test. A Bonferroni correction was applied across the five omnibus tests (*α* = .010); all remained significant (*p*s < .001). Games–Howell post hoc tests were used for life satisfaction, anxiety, and depression, Dwass–Steel–Critchlow–Fligner comparisons for continuous NSSI, and *z*-tests for proportions with Bonferroni-adjusted *p*-values for repetitive NSSI.

Cluster 6 did not differ significantly from Clusters 4 or 5 on any continuous outcome (all *p*s > .05) but showed poorer outcomes than Clusters 1–3 (all *p*s ≤ .020). The same pattern emerged for repetitive NSSI: Cluster 6 did not differ significantly from Clusters 4 or 5 (all *p*s ≥ .113) but had a higher prevalence than Clusters 1–3 (all *p*s ≤ .003).
